# Cardiac Lipoma: An Uncharacteristically Large Intra-Atrial Mass Causing Symptoms

**DOI:** 10.1155/2018/3531982

**Published:** 2018-02-06

**Authors:** Fahad Syed Naseerullah, Hemangkumar Javaiya, Avinash Murthy

**Affiliations:** ^1^SSM Health Good Samaritan Hospital, Good Samaritan Way, Mount Vernon, IL 62864, USA; ^2^Southern Illinois Heart and Vascular Center, 4115 S. Water Tower Place, Mount Vernon, IL 62864, USA

## Abstract

Primary tumours of the heart are often encountered in clinical practice. Different autopsy series estimate the incidence to be anywhere from 0.001% to 0.19%. Cardiac lipoma is a rare type of tumour of the heart and pericardium. It comprises approximately 10–19% of all cardiac tumours. We present a case of a large cardiac lipoma in a fifty-year-old female. She presented with sharp chest pains, palpitations, and dizziness. Acute coronary syndrome was ruled out. A transthoracic echocardiogram showed an abnormal, large, fixed right atrial mass. The mass was noted to be occupying most of the right atrium. It was excised due to its large size and persistent symptoms. On pathophysiology, the mass was definitively diagnosed to be an 80 mm × 70 mm cardiac lipoma. Postoperatively, the patient did well with resolution of her symptoms. This case provides evidence that even large, invasive, symptomatic cardiac lipomas can be successfully resected with good outcomes.

## 1. Introduction

Primary tumours of the heart are rarely encountered in clinical practice. Different autopsy series estimate the incidence to be anywhere from 0.001 to 0.19% [1, 2]. Cardiac lipomas comprise approximately 10–19% of all primary tumours of the heart and pericardium and make up 8.4% of all the benign ones [2, 3].

Most cardiac lipomas remain small and do not cause symptoms, or if they do, they may remain asymptomatic for a long while [1, 4]. However, like the case here demonstrates, at times they can grow large and cause significant symptoms. When symptomatic, patients with cardiac lipoma can have various presentations, depending on the size and location of the tumour [3]. No clear guidelines have been established due to the low incidence of cardiac lipomas, but resection has been reported to be effective.

## 2. Case Presentation

A fifty-year-old female, presented to the emergency room, with central, sharp chest pain for about two hours. The pain radiated to the neck and caused left arm tingling. It was associated with nausea and diaphoresis. Independent of the chest pain, the patient complained of frequent palpitations for a few months which now were getting more frequent and prolonged. The patient also complained of episodes of dizziness for the last few days. These episodes were preceded by palpitations. Her past medical history included hypertension and an episode of transient ischemic attack. Her only home medication was aspirin 81 mg daily. Her physical examination was unremarkable.

**Figure 1:**
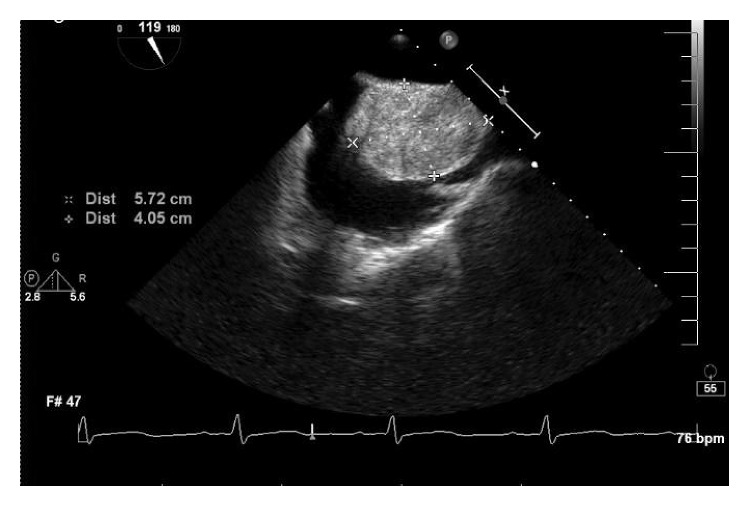
Transoesophageal echocardiogram showing the right atrial mass of 40 mm × 57 mm.

The patient had an EKG and serial troponins which were within normal limits. A transthoracic echocardiogram was obtained for further workup. This showed an abnormal, large right atrial mass. For further characterization of the mass and to limit the acoustic window, a transoesophageal echocardiogram was obtained. It demonstrated a 40 mm × 57 mm, large solid, fixed mass with smooth contours occupying most of the dilated right atrium (Figure 1). The mass appeared to be attached to the inferior part of the right atrial septum. Coronary angiography was performed which did not show any obstructive coronary artery disease.

**Figure 2:**
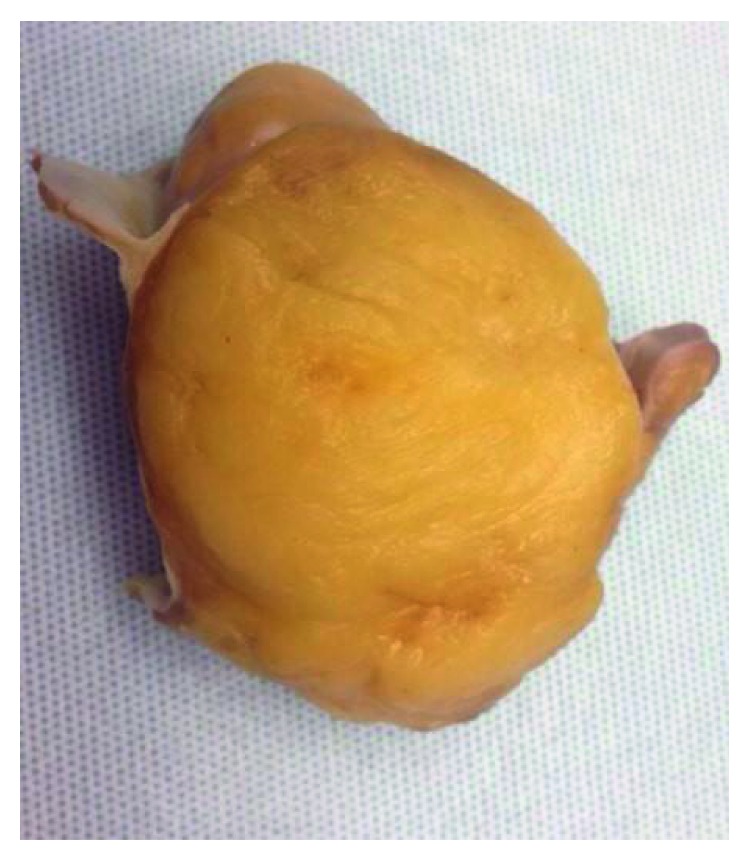
The surface of the specimen is lined by smooth endocardial and epicardial tissue. The cut surface displays a yellow, lobulated appearance without haemorrhage, necrosis, fleshy change, or calcification.

A cardiothoracic surgery consultation was obtained for the patient. Since the tumour was well characterized on the TEE, any further imaging was not thought to be necessary. Given the patient's significant symptoms and the large size of the mass, surgical resection was planned. A well-circumscribed, right atrial mass of 80 mm × 70 mm was removed (Figure 2). It was found to be involving the endocardium of the atrial septum, the right atrial wall, and the superior vena cava-right atrium junction. The atrial septum and the roof required reconstruction which was done with two Gore-Tex cardiovascular patches. In addition, reconstruction of the superior vena cava-right atrium junction was performed using interposition graft.

The patient did well postoperatively. She had resolution of her symptoms. She did have intermittent asymptomatic junctional bradycardia which was monitored. We chose to anticoagulate her for three months due to the sutures that were placed inside the atrium.

At her six-month follow-up, the patient appeared to be doing well. She, however, remained in junctional rhythm and was starting to become symptomatic with complaints of generalized weakness. A permanent pacemaker was therefore implanted without any complications.

## 3. Discussion

Cardiac lipomas are rare, benign tumours that usually remain small, asymptomatic, and are found incidentally on autopsy [1, 2]. This is a rare case where the tumour was large and was determined to be causing significant symptoms. Since surgical resection has been reported to have good outcomes, inclusion of cardiac lipoma in differentials is imperative.

**Figure 3:**
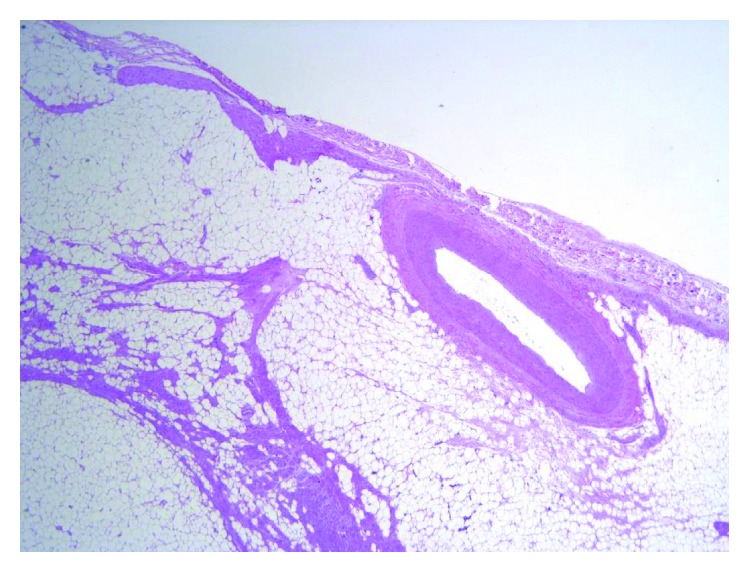
Haematoxylin and eosin stained, 20x magnification. The section includes the epicardial surface and a patent coronary artery. The atrial wall is replaced by proliferation of benign adipocytes, which displace and infiltrate the myocardium.

**Figure 4:**
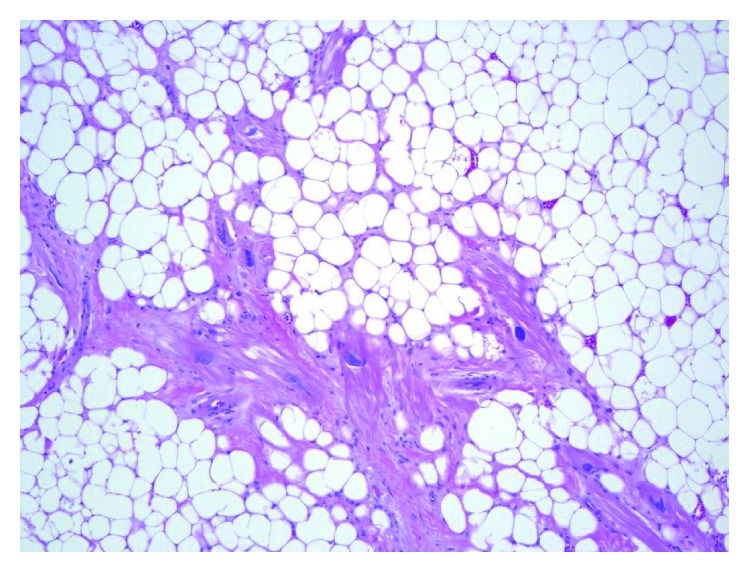
Haematoxylin and eosin stained, 100x magnification. The adipocytes are monotonous with small, marginalized nuclei. No cytologic atypia or lipoblasts are present. The cardiac myocytes display reactive nuclear features and degenerative cytoplasmic changes.

Lipomas in general are most commonly seen to have translocations involving chromosome 12 [5]. The etiology of cardiac lipomas remains undetermined. They are benign tumours of the heart that are mainly composed of mature adipocytes without any cellular atypia (Figures 3 and 4) [1–4]. They are usually well-encapsulated masses [1, 3]. Grossly, they appear as yellow fat divided by fine fibrous trabeculae [4]. The capsule is a major differentiation point between cardiac lipoma and lipomatous hypertrophy. The latter is usually an asymptomatic continuation of the epicardial fat, and when arising from the interatrial septum, it usually spares the fossa ovalis [2]. Unlike, cardiac lipomas which do not have any clear associations, lipomatous hypertrophy is seen more commonly in older, obese people and in females [3].

Cardiac lipomas can originate from all the three cardiac tissue layers: most commonly being subendocardial, followed equally by subpericardial, and finally myocardial in location [3]. When intracardiac, they are most commonly found in the right atrium and the left ventricle but can be seen in almost any other location of the heart [1, 4]. They usually have a broad base or peduncle of attachment and are less mobile compared to other benign tumours of the heart [4, 6].

Most cardiac lipomas do not cause symptoms or they may remain asymptomatic for a long time [1, 4]. They mas cause arrythmias, or symptoms due to invasion or mass effect [1, 2]. They can cause a whole spectrum of symptoms depending on their size and location including dyspnea, chest pain, arrhythmias, syncope, and stroke-like symptoms [2]. The mechanisms driving these symptoms include direct obstruction of intracardiac blood flow, dysfunction of cardiac valves, obstruction of the superior and inferior vena cava, and even phrenic nerve involvement [4]. Systemic or pulmonary thromboembolisms and pericardial effusion with or without tamponade have also been reported in the literature [3]. Apart from this, sudden death caused by cardiac lipoma has also been reported [7]. In our case, the patient had a history of TIA and presented with chest pain, palpitations, and dizziness which were probably associated with the growing right atrial tumour.

Due to its easy availability and noninvasive nature, transthoracic echocardiography is the most common initial investigation to define the presence, extent, and location of cardiac tumours [2, 4]. Transoesophageal echocardiography has the benefit of not being limited by acoustic windows [6]. Echocardiography has a 90% sensitivity and a 95% specificity in detecting cardiac tumours and allows a dynamic evaluation of the mass with characterization of their physiologic consequences [3]. Both cardiac computerized tomography (CCT) and cardiac magnetic resonance (CMR) can help identify the presence of fat and so can be used to correctly diagnose cardiac lipoma [6]. They can characterize the composition of the tumour and hence aid in the differentiation between various types of cardiac tumours [3]. In addition, coronary angiography prior to surgery is recommended to exclude any coronary artery involvement and to identify a possible coronary source of the blood supply to the tumour [2–5].

Due to the rare occurrence of cardiac lipomas, no guidelines have been established, but there is consensus that surgical resection should be attempted in all symptomatic patients [1, 2]. Complete excision along with the capsule is preferred to prevent local recurrence, even though the rate of recurrence of cardiac lipoma appears to be quite low despite subtotal resection [4]. Management of asymptomatic patients with incidentally found tumours remain less straightforward. Close monitoring is prudent in such patients. Depending on the size and rate of growth of the tumour, surgical intervention may be considered.

Cardiac lipoma is quite a rare occurrence. In addition, it has a wide spectrum of presentation ranging from being asymptomatic to causing heart failure and to even causing sudden death. Therefore, a high index of suspicion is necessary for a prompt diagnosis. The advancements in imaging technology have made preoperative diagnosis more accurate. In symptomatic patients with this benign tumour, surgery provides a curative rate of 95%, and the overall prognosis is considered quite good [3].
